# Engeletin alleviates depression‐like phenotype by increasing synaptic plasticity via the BDNF‐TrkB‐mTORC1 signalling pathway

**DOI:** 10.1111/jcmm.17975

**Published:** 2023-10-06

**Authors:** Yangyang Xu, Jie Zhang, Linyao Yu, Wei Zhang, Yingtian Zhang, Yaoqin Shi, Shuping Zhang, Chunmei Li, Jingwei Tian

**Affiliations:** ^1^ School of Pharmacy, Key Laboratory of Molecular Pharmacology and Drug Evaluation (Yantai University), Ministry of Education, Collaborative Innovation Center of Advanced Drug Delivery System and Biotech Drugs in Universities of Shandong Yantai University Yantai P. R. China; ^2^ Department of Pharmacy Binzhou Medical University Hospital Binzhou P. R. China; ^3^ Department of Radiology Binzhou Medical University Hospital Binzhou P. R. China; ^4^ College of Basic Medicine Binzhou Medical University Yantai P. R. China

**Keywords:** BDNF‐TrkB‐mTORC1 signalling pathway, chronic restraint stress, engeletin, major depressive disorder, prefrontal cortex, synaptic plasticity

## Abstract

Major depressive disorder (MDD) is a severe mental disorder associated with high rates of morbidity and mortality. Current first‐line pharmacotherapies for MDD are based on enhancement of monoaminergic neurotransmission, but these antidepressants are still insufficient and produce significant side‐effects. Consequently, the development of novel antidepressants and therapeutic targets is desired. Engeletin, a natural *Smilax glabra rhizomilax* derivative, is a compound with proven efficacy in treating ischemic stroke, yet its therapeutic effects and mechanisms for depression remain unexplored. The effects of engeletin were assessed in the forced swimming test (FST) and tail suspension test (TST) in mice. Engeletin was also investigated in the chronic restraint stress (CRS) mouse model of depression with fluoxetine (FLX) as the positive control. Changes in prefrontal cortex (PFC) spine density, synaptic plasticity‐linked protein expressions and the brain‐derived neurotrophic factor (BDNF)‐tyrosine kinase B (TrkB)‐ mammalian target of rapamycin complex 1 (mTORC1) signalling pathway after chronic stress and engeletin treatment were then investigated. The TrkB and mTORC1 selective inhibitors, ANA‐12 and rapamycin, respectively, were utilized to assess the engeletin's antidepressive mechanisms. Our data shows that engeletin exhibited antidepressant‐like activity in the FST and TST in mice without affecting locomotor activity. Furthermore, it exhibited efficiency against the depression of CRS model. Moreover, it enhanced the BDNF‐TrkB‐mTORC1 pathway in the PFC during CRS and altered the reduction in dendritic spine density and levels of synaptic plasticity‐linked protein induced by CRS. In conclusion, engeletin has antidepressant activity via activation of the BDNF‐TrkB‐mTORC1 signalling pathway and upregulation of PFC synaptic plasticity.

## 1 INTRODUCTION

Depression comprises multiple syndromes and is manifested with substantial and persistent mood disorders.^1^ It is a leading cause of total disability and increases the economic burden. Although antidepressants have been clinically utilized for decades, the molecular and cellular pathways affecting therapeutic actions are poorly elucidated.^2^, ^3^ Recently, the research hotspot has been the identification of non‐monoamine‐based antidepressants. Most clinically used are antidepressants synthetic compounds, such as selective 5‐hydroxytryptamine reuptake inhibitors (SSRIs).^4^ Although the currently applied treatment regime alleviates depression, the symptoms are not completely resolved in about 50% of the cases and the remission rates in individuals who failed initial treatment are even worse.^2^, ^5^ Therefore, antidepressants with enhanced efficacy and fewer adverse events are required.

The 5‐hydroxytrypta‐minergic system dysfunction causes depression and is mostly treated via SSRIs. Recently, a leading depression hypothesis suggested that synaptic plasticity and neurotrophic factors essentially mediate behavioural responses to antidepressants.^6^, ^7^ Antidepressants modulate the brain‐derived neurotrophic factor (BDNF) and induce antidepressant‐like influence in short‐term behavioural depression models.^8^, ^9^ Furthermore, stress‐induced negative cellular functional and morphological alterations in the prefrontal cortex (PFC) are consistent with the levels of essential receptors and proteins associated with synaptic plasticity.^10^, ^11^ These studies indicate that BDNF signalling pathway stimulation and PFC synaptic plasticity are novel targets against depression.

**FIGURE 1:**
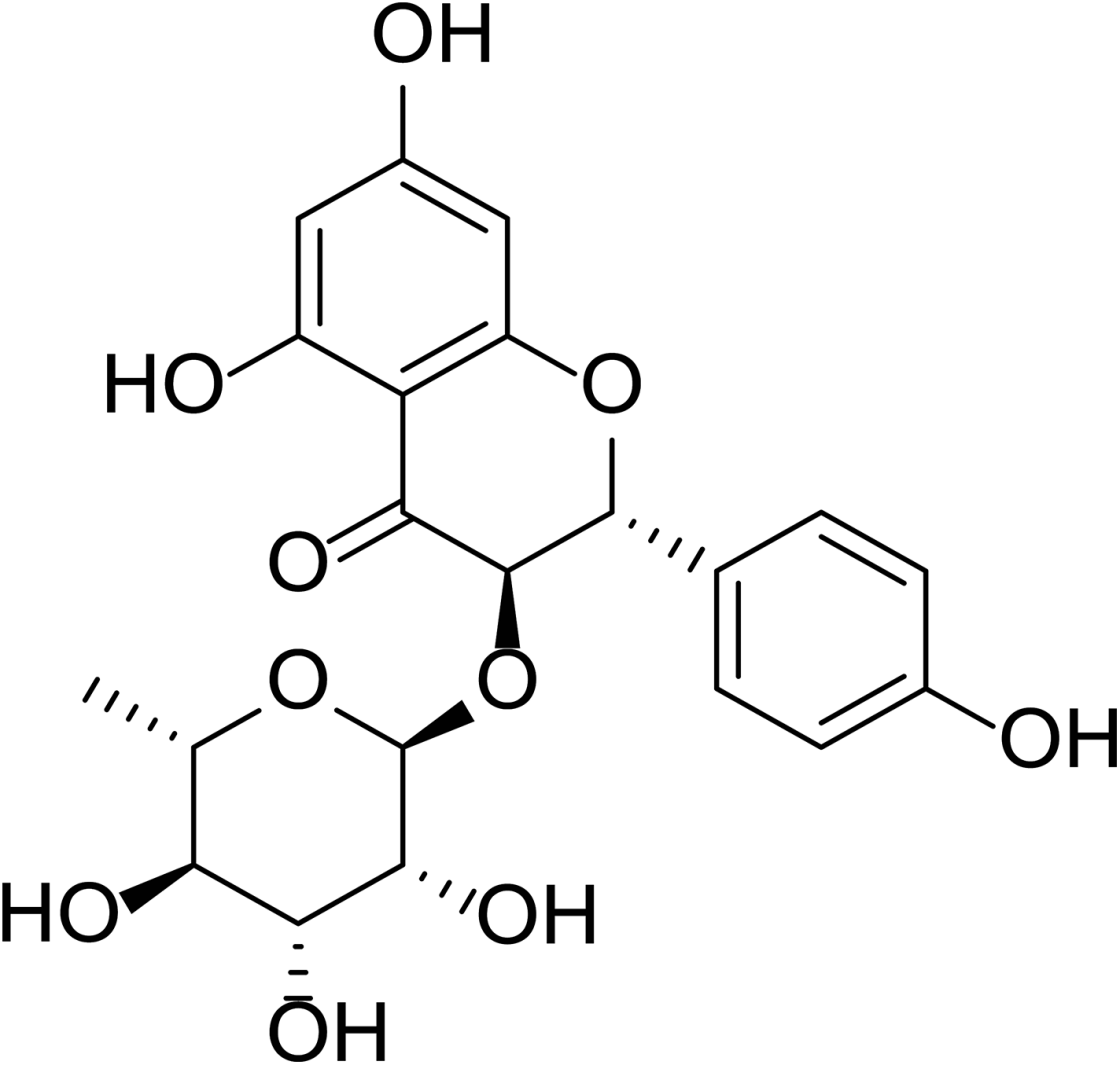
The chemical structure of engeletin.

Dihydrokaempferol 3‐O‐a‐L‐rhamnoside, also called engeletin (Figure 1), is among the most abundant and markedly active components of *Smilax glabra rhizomilax*. The literature suggests that engeletin has neuroprotective properties and minimal toxicity.^12^ Various neurotrophic and neuroprotective activities of engeletin have been indicated, such as its efficiency against ischemia/reperfusion and Alzheimer's disease, reactive oxygen species production suppression and its anti‐oxidant and anti‐inflammatory properties.^13^, ^14^, ^15^ Furthermore, engeletin is widely utilized for treating acute surgical infections, rheumatic arthritis and cancers. It also protects and treats acute lung injury via peroxisome proliferator‐mediated receptor gamma. However, engeletin's antidepressant effect remains undetermined.

This investigation aimed to elucidate the antidepressant effects of pure engeletin using various depression models, including the tail suspension test (TST), forced swimming test (FST), and chronic restraint stress (CRS). Moreover, the mechanisms underlying these antidepressant influences were assessed.

## 2 MATERIALS AND METHODS

### 2.1 Animals

The C57BL/6 (age = 8 w, male and weighed = 20–22 g) mice were acquired from Jinan Pengyue Experimental Animal Breeding Co., Ltd., and housed in controlled temperature and humidity environments with a 12‐h light (from 7:00 to 19:00) cycle. The animals were provided ad libitum chow and water. All mice protocols followed the Guide for the Care and Use of Laboratory Animals by the National Institutes of Health, USA, and were authorized by the Laboratory Animals Care and Use Committee of Yantai University. For deep anaesthesia, isoflurane (2–5 vol %) was administered nasally, and for euthanization, cervical dislocation protocol was applied, making sure to minimize suffering.

### 2.2 Drugs and reagents

Engeletin (>99% pure with a molecular weight of 434.39) was provided by SenBeiJia Biological Technology (China). Fluoxetine hydrochloride (FLX) (Sigma‐Aldrich) was prepared using 0.5% sodium carboxymethylcellulose (SCMC). For intraperitoneal administration (i.p.): TrkB and selective mTOR inhibitors, ANA‐12 and rapamycin (MedChem Express), in concentrations 0.5 and 5 mg/kg, were dissolved in 0.5% SCMC, respectively.

### 2.3 Behavioural measurements

#### 2.3.1 Forced swimming test

This experiment was carried out per our previous study protocol and other literature, with slight modifications.^5^ Briefly, each mouse was put in a glass cylinder (diameter = 10 cm, height = 25 cm) filled with water (height = 10 cm at 25 ± 1°C), which was refreshed per trial. All mice were forced to swim for 6 min, and during the last 4 min, the immobility time was recorded.

#### 2.3.2 Tail suspension test

After the tail suspension, the total immobility time was assessed by following the protocol in the literature.^16^ Briefly, adhesive tape was stuck to the tail's tip about 1 cm below to suspend mice for 6 min, 50 cm above the floor. In the last 4 min, the immobility time was measured. Immobility was defined when mice were hanging completely motionless and passively. Mice who climbed their tails were excluded from the assay.

#### 2.3.3 Open field test

In rodents, increased locomotor decreases immobility duration in the TST and FST and might cause false‐positive data (Bourin et al., 2001). An OFT excludes this possibility; therefore, this test was performed as stated in previous reports. Each mouse was tested in an open field test (OFT) apparatus (100 × 100 × 45 cm, illuminated with a 50‐W red bulb on the roof) for 10 min. The number of central and peripheral squares crossed by each animal during this time was elucidated by an observer blinded to mice grouping under dim light.

#### 2.3.4 Sucrose preference test

This test was carried out in the light. Before the test, each 24 h water‐deprived mouse was given two bottles: (1) 1% sucrose solution and (2) tap water. Sucrose preference test (SPT) was performed for 4 h, and the liquid consumption was assessed by subtracting the bottle weights. Furthermore, prior to testing, mice were given a 48‐h sucrose preference training and sucrose preference (%) was elucidated as follows: Sucrose solution consumption/total fluid consumed × 100.

### 2.4 Chronic restraint stress

The CRS was performed by following the literature‐suggested protocol.^17^ Briefly, stressed mice were subjected to CRS for 4 weeks, 3 h daily, from 9 to 12 a.m. 50 mL of conical plastic tubes with vent holes was utilized for restraint stress. The mice's depressive‐like behaviours were assessed via SPT, FST, TST and OPT.

### 2.5 Golgi staining

The FD Rapid GolgiStain kit (FD Neuro Technologies) was utilized for brain sample preparation, per the kit's protocol. 150 μm thick coronal sections were sliced via a Leica freezing microtome, inoculated in FD Solution C droplets on gelatin‐laminated slides (FD NeuroTechnologies, P0101), mounted, dried overnight at ambient temperature, immersed in the working solution and then by using absolute ethanol at 50%, 75% and 95% concentration the sections were dehydrated. Then the slices were cleared via Xylene and covered using a cover slip. The dendritic spine (DS) was visualized via a Nikon Eclipse Ci‐L microscope.

### 2.6 RNA extraction and real‐time PCR

The whole RNA was acquired from the PFC tissue of mice from each group (*n* = 3/group) with the help of TRIzol (Invitrogen, CA, United States) per the kit's guide. SPARK script II RT Plus Kit (Spark Jade, Shandong, China) was utilized for cDNA preparation, and GAPDH was used for normalization. For each gene, the protocol was replicated thrice before the quantitative gene expression assessment via the 2^−ΔΔ^CT protocol. All primers were acquired from Biotech Biotechnology Inc (Biotech), and their sequences are given below:

BDNF: forward: 5′‐CAGGACAGCAAAGCCACAAT‐3′ and reverse: 5′‐GCCTTCATGCAACCGAAGTA‐3′;

GAPDH: forward: 5′‐CTCTCTGCTCCTCCCTGTTC‐3′ and reverse: 5′‐CCGACCTTCACCATTTTGTC‐3′.

### 2.7 Western blots

The brain tissues were sampled from the sacrificed mice immediately after the behavioural tests were carried out. Using the radioimmunoprecipitation assay buffer augmented with enzyme inhibitors, the PFC was lysed on ice. The tissues were immediately homogenized for western blotting using the primary antibodies stated below: rabbit anti‐mTOR (ab134903, 1:1000), rabbit anti‐BDNF (ab108319, 1:1000), rabbit anti‐GLUR1 (ab183797, 1:1000), rabbit anti‐phosphorylated‐mTOR (phospho S2448, ab109268, 1:1000) and rabbit anti‐PSD95 (ab238135, 1:1000) (all from Abcam Inc.), whereas mouse anti‐GAPDH (AF0006, 1:1000) was acquired from Beyotime Biotechnology Inc.). Horseradish peroxidase‐linked goat anti‐rabbit IgG (H + L) was utilized as the secondary antibody (Proteintech; 1:3000). The positive immune reaction on blots was observed using Super Signal West Pico Chemiluminescent Substrate (Thermo Fisher Scientific Inc.). The acquired data were normalized with the relative GAPDH density.

## 3 STATISTICAL ANALYSIS

All the values are indicated as mean ± standard error of the mean (SEM). The variabilities in the mean values were elucidated by one‐or two‐way anova, as appropriate. Statistically important values were defined as those with *p* < 0.05.

## 4 RESULTS

### 4.1 Antidepressant impact of engeletin in the FST and TST mice

**FIGURE 2:**
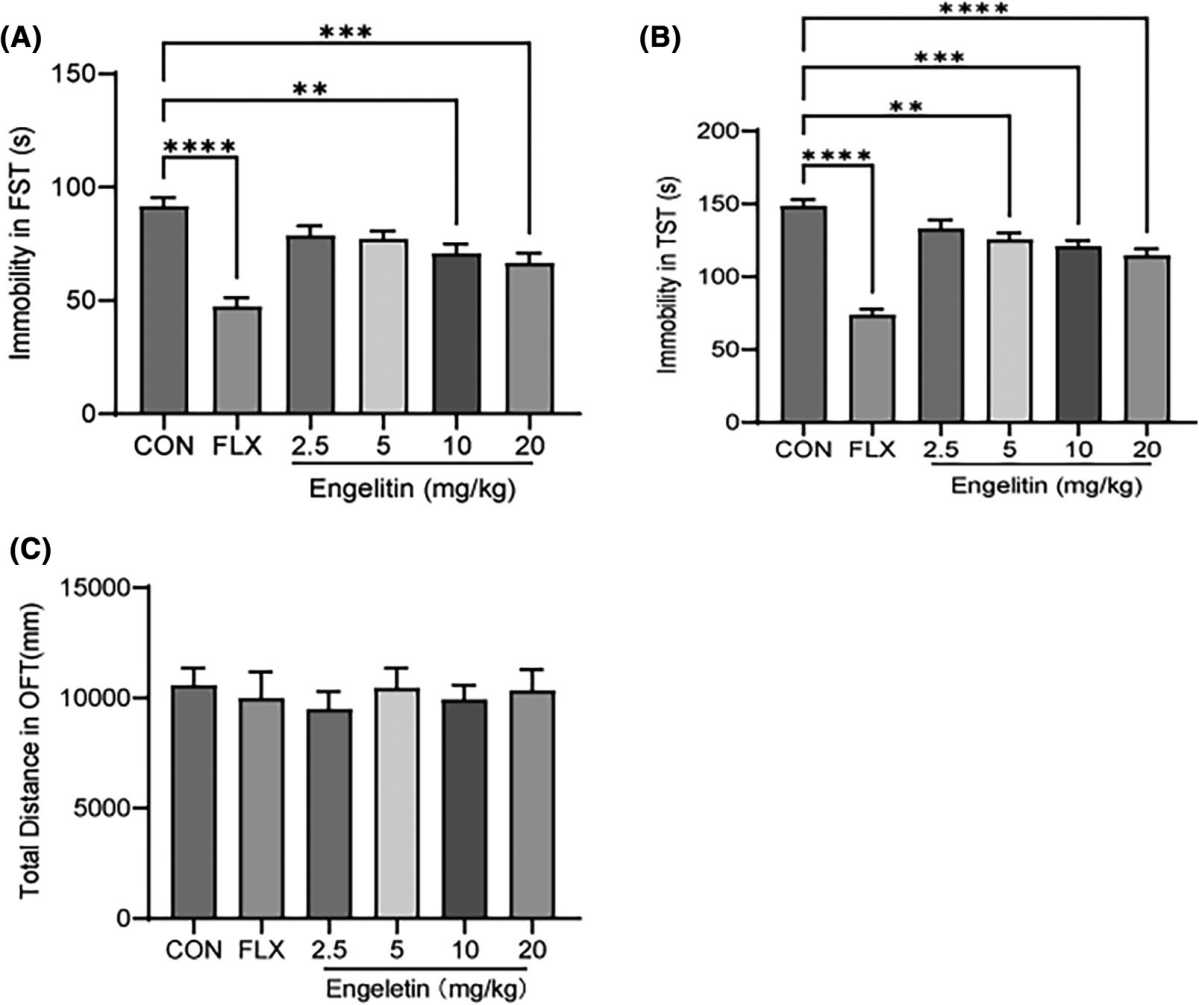
Engeletin produces an antidepressant‐like effect in the FST and TST. The mice received a single dose of saline (CON), FLX (10 mg/kg) or engeletin (2.5, 5, 10, 20 mg/kg). After 1 h of treatments, the behavioural tests were performed. (A) Engeletin dose‐dependently reduced the immobility time in the FST. (B) Engeletin dose‐dependently reduced the immobility time in TST. (C) Engeletin indicated no effect on spontaneous locomotor function in the OFT. The data are depicted as mean ± SEM (*n* = 12). *p < 0.05, **p < 0.01, ***p < 0.001, ****p < 0.0001 substantially different from control; one‐way anova followed by post hoc LSD test.

The TST and FST are largely applied behavioural tests in mice for elucidating the potential antidepressant‐like property because of their increased validity for predicting AD‐like activity. Therefore, the possible antidepressant properties of engeletin were first assessed by FST. Engeletin (2.5, 5, 10, 20 mg/kg) was given intragastrically (i.g.). Fluoxetine (FLX, 10 mg/kg, i.g.) was utilized as a positive control. It was revealed that a single engeletin dose had a strong antidepressant effect during FST (Figure 2A). After performing one‐way anova on the data using drug treatment as the factor, its significant main effect was indicated. Furthermore, post hoc analysis suggested that enhanced engeletin doses (2.5–20 mg/kg) reduced the immobility period in the FST, dose‐dependently, which was substantially alleviated at the increased 10 and 20 mg/kg doses. FLX also markedly decreased the immobility time, consistent with previous data.

The dose–response assays were carried out to elucidate engeletin's antidepressant properties via the TST (Figure 2B). The significant drug treatment effects were revealed. Engeletin (5–20 mg/kg, i.g.) dose‐dependently reduced the immobility time in the TST and FST. According to the post hoc analysis, engeletin‐ (5–20 mg/kg) and FLX‐treated mice had markedly decreased immobility time than saline‐treated mice.

To eliminate the possibility that this reduction was because of enhanced spontaneous activity, the naive mice (treated as above) were subjected to OFT for 10 min. No difference in the total distance was observed between the groups (Figure 2C), and anova indicated no impact of engeletin treatment. Acute engeletin dose did not affect locomotor function, indicating that the reduced immobility post‐engeletin treatment in the two tests was not because of locomotor hyperactivity.

### 4.2 Engeletin's effects on body weight gain and depressive‐like behaviours of stressed mice

**FIGURE 3:**
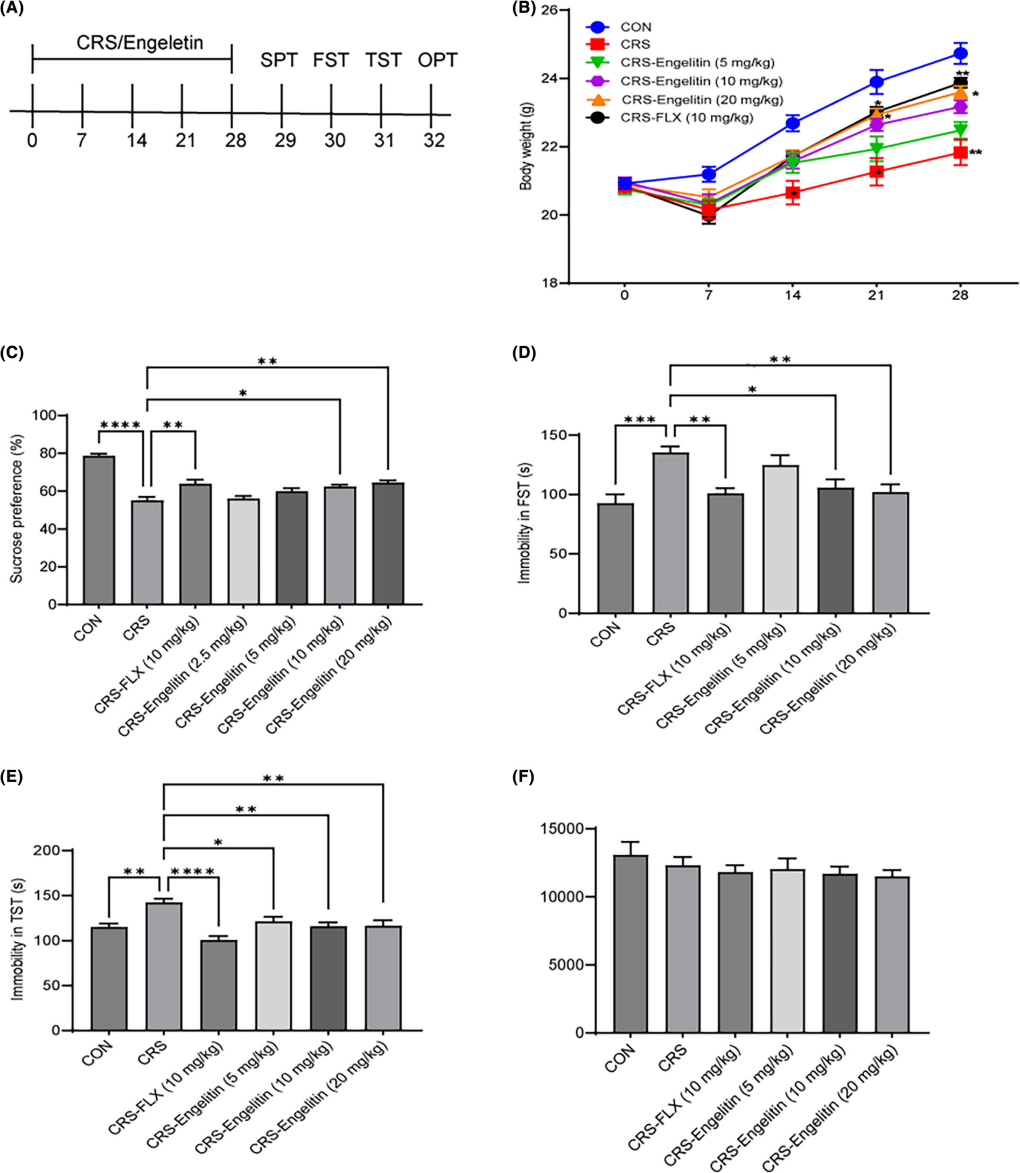
Engeletin reverses CRS‐induced depressive‐like behaviours in mice. (A) Experimental protocol timeline. During the investigation, the mice's body weight was assessed every 7 days and behavioural tests, including TST, FST, SPT and OFT, were carried out on each mouse/group at the end of the experiment. (B) Influence of engeletin on the CRS mice's body weight alterations. (C) The sucrose preference rate in the SPT. (D) The FST immobility time. (E) The TST immobility time. (F) The total distance travelled in the OFT. The data are indicated as mean ± SEM (*n* = 11–12). *p < 0.05, **p < 0.01, ****p < 0.0001, two‐way anova followed by post hoc Bonferroni's test.

To further characterize the antidepressant effects of engelitin, we employed CRS, which is currently regarded as one of the most predictive animal models of depression.^18^, ^19^ No substantial alterations were observed in each group's mice body weight before the experiment; however, after inducing CRS, the weight of mice in differrent groups altered (Figure 3B). The model group mice's body weight notably reduced after 14 days of CRS procedures than the control mice. Whereas the body weight of the engeletin‐treated mice started increasing markedly on the 21st day, their mean weight on the 21st and 28th days was substantially higher than that in the non‐treated model mice (Figure 3B). CRS reduced the sucrose consumption in the mice more than in the control (Figure 3C), which was substantially reversed by increased engeletin systemic administration, suggesting that engeletin might enhance hedonic states in mice.

Further behavioural assays indicated that 28 days of engeletin treatment notably alleviated the CRS‐promoting effects on mice immobility in the FST and TST (Figure 3D,E). Moreover, no marked changes were observed in the locomotor function between each group (Figure 3F). These data revealed that engeletin could reverse the CRS‐mediated depressive‐like behaviours in mice.

### 4.3 Engeletin reverses the CRS‐induced decrease in dendritic spine density and synaptic plasticity

**FIGURE 4:**
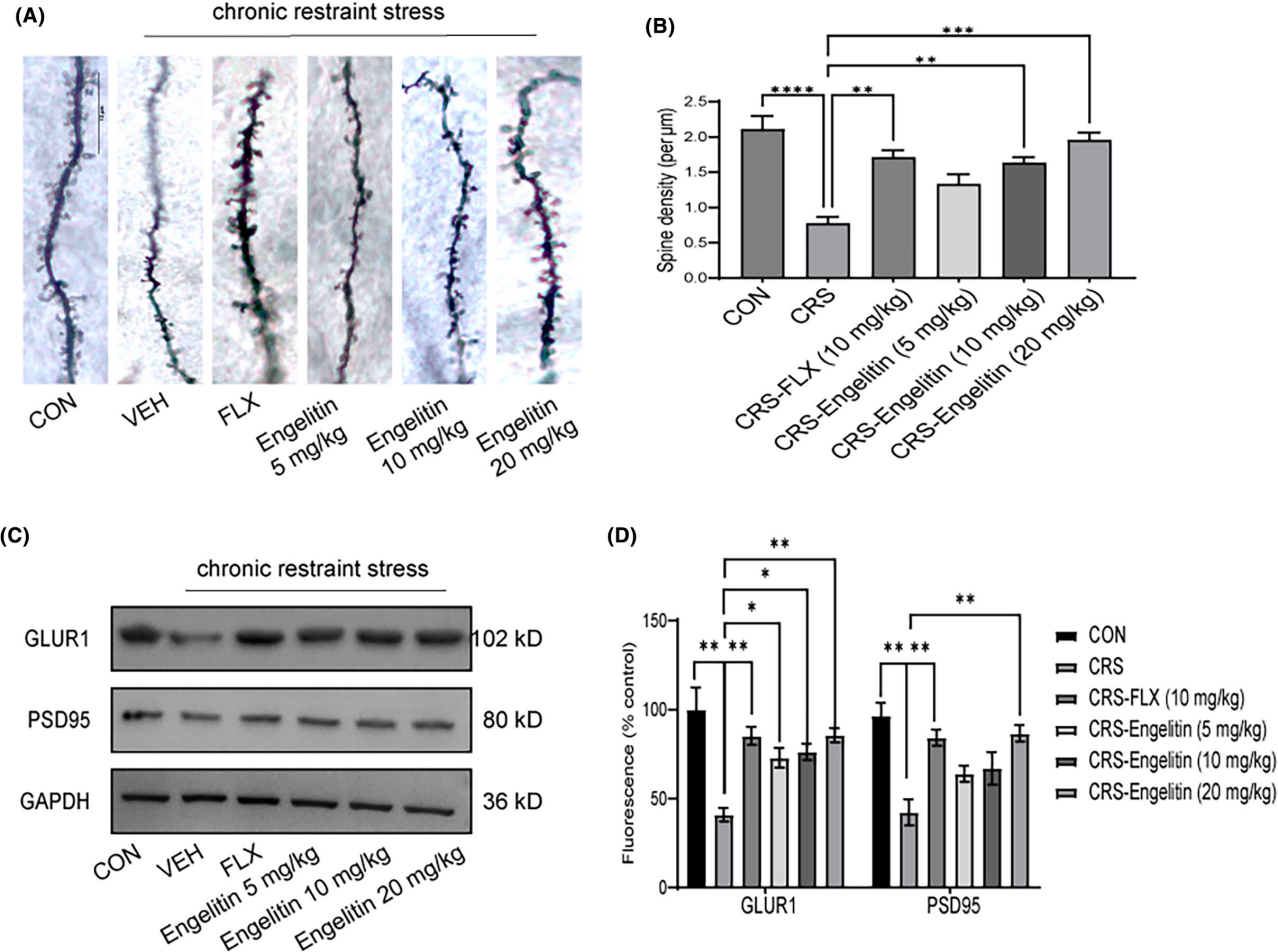
Engeletin reverses the change in PFC spine density and the decreased protein expression of PFC synaptic plasticity induced by CRS. (A) Representative photomicrograph of a Golgi‐stained PFC pyramidal neuron from each mouse/group. Scale bar = 10 mm. (B) The DSs/10 μm were quantified by Golgi staining. (C, D) Western blotting indicated that the engeletin regimen reversed the CRS‐mediated reduction of PFC's GLUR1 and PSD95 proteins. Data are depicted as mean ± SEM (*n* = 3). *p < 0.05, **p<0.01, ***p<0.001, ****p<0.0001. Two‐way anova followed by post hoc Bonferroni's test.

Much evidence suggests the synaptic plasticity dysregulation in depression aetiology, causing neuronal atrophy and synaptic weakening in critical brain regions (hippocampus and PFC).^20^, ^21^ Here, synaptic plasticity was assessed by PFC samples, western blotting and Golgi staining. Figure 4A,B indicates distal dendrites in PFC. The DS density was notably reduced in CRS mice than the controls, and engeletin alleviated this reduction, similar to FLX. These results suggest that engeletin can rescue CRS‐mediated DS density decrease. Figure 4C,D indicates that the expression of GLUR1 and PSD‐95 in the model mice's PFC were markedly downregulated than the control mice. Whereas engeletin (20 mg/kg) and FLX treatments markedly upregulated these protein levels more than the model mice. Moreover, GLUR1 protein analysis revealed that it was substantially enhanced in the PFC of all treatment groups than in model mice.

### 4.4 Chronic engeletin treatment alleviates the CRS‐mediated BDNF‐TrkB signalling pathway reduction in the PFC

**FIGURE 5:**
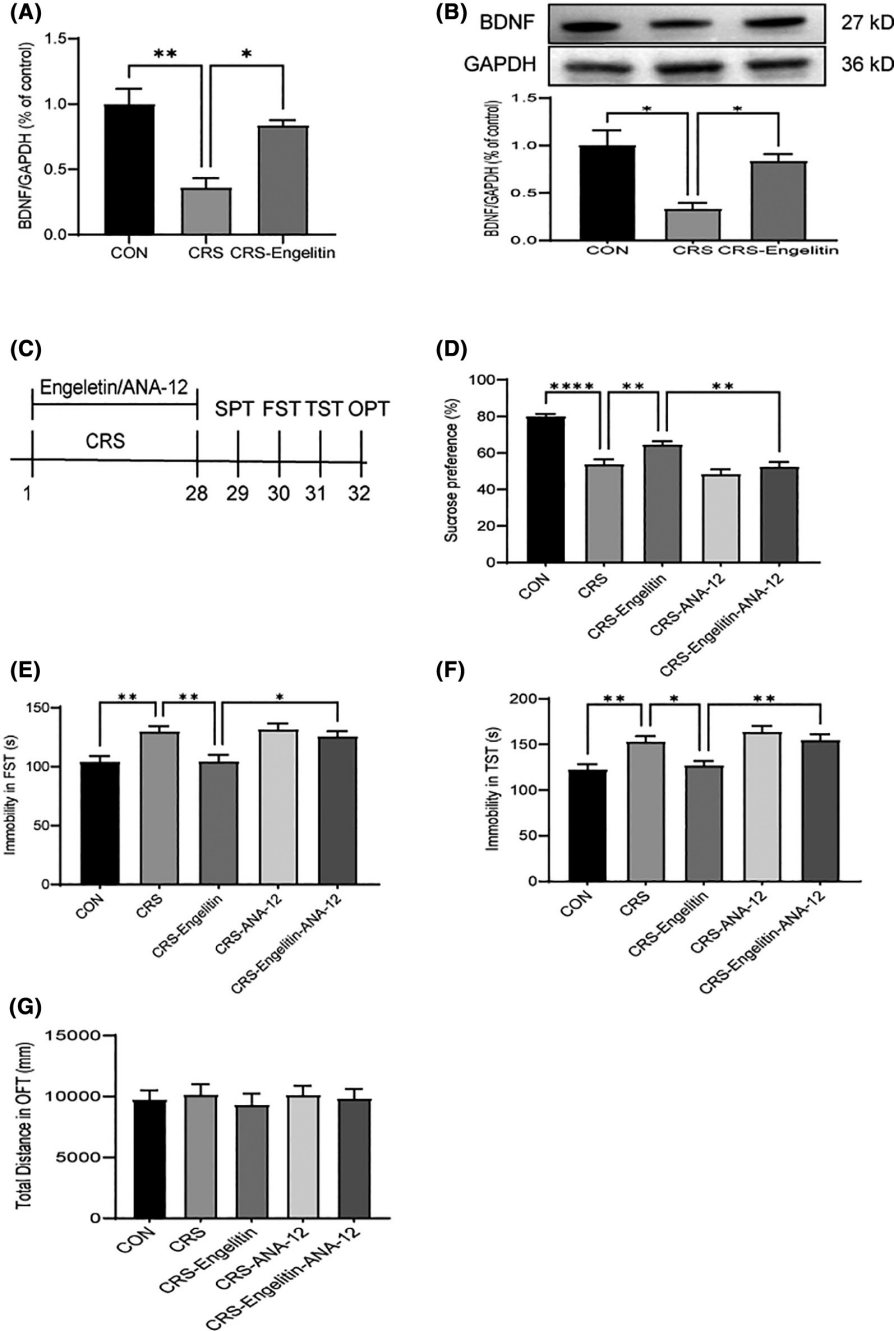
Chronic engeletin treatment increases BDNF signalling in the PFC of stressed animals. (A) RT‐PCR data indicated that 28 days of chronic engeletin treatment reversed the reduction of BDNF mRNA in CRS‐mediated PFC (*n* = 3). (B) Western blotting indicated that the engeletin regimen reversed the CRS‐mediated reduction of PFC's BDNF protein (*n* = 3). (C) Experimental protocol timeline. (D) In the CRS model, engeletin + ANA‐12 mice had reduced sucrose preference than engeletin mono‐treatment mice (*n* = 12). (E, F) In the CRS model, engeletin + ANA‐ 12 mice exhibited increased immobility time than engeletin mono‐treatment mice in the TST and FST (*n* = 12). (G) Neither ANA‐12 nor engeletin substantially affected the locomotor function of naive mice in the OFT (*n* = 12). Data are indicated as mean ± SEM. *p < 0.05, **p<0.01, ****p<0.0001. Two‐way anova followed by post hoc Bonferroni's test.

Synaptic plasticity is regulated by modulating multiple signalling pathways, and disruptions of major pathways increase the vulnerability to depression, such as neurotrophic factors loss. BDNF is packaged and released from postsynaptic spines and acts in an autocrine cell autonomous manner to enhance spine maturation and number.^22^, ^23^ To elucidate the mechanisms associated with engeletin's antidepressant effects, BDNF expression was assessed, which is essential for neuronal survival and brain neurogenesis and has been linked with current depression‐related theories. Therefore, after CRS, the mRNA levels of BDNF (expressed as a ratio of GAPDH expression) in the PFC were measured. Figure 5A summarizes the data. Furthermore, the modifying BDNF protein levels parallelled these alterations were identified. As Figure 5B depicts, CRS and drug administration had a main effect. While CRS reduced the expression of BDNF protein, engeletin markedly increased its levels at 20 mg/kg, consistent with the increased BDNF mRNA levels.

BDNF and its specific receptor TrkB are essentially linked with depression pathophysiology and mechanisms of antidepressants. To assess if BDNF‐TrkB signalling is lined with engeletin's antidepressant effects, ANA‐12, a potent pharmacological TrkB inhibitor, was administered in mice (0.5 mg/kg, i.p.) 30 min before engeletin (20 mg/kg, i.g., daily) treatment. It was observed that engeletin and ANA‐12 treatment suppressed the sucrose preference in the CRS mice (Figure 5D). Furthermore, ANA‐12 drastically inhibited the engeletin‐mediated decreased immobility period in TST and FST (Figure 5E,F). These data suggest that ANA‐12 antagonizes the antidepressant properties of engeletin. Together, the BDNF‐TrkB signalling pathway is essential for the antidepressant impact of engeletin.

### 4.5 mTOR is necessary for engeletin to promote synaptic plasticity to play an AD role

**FIGURE 6:**
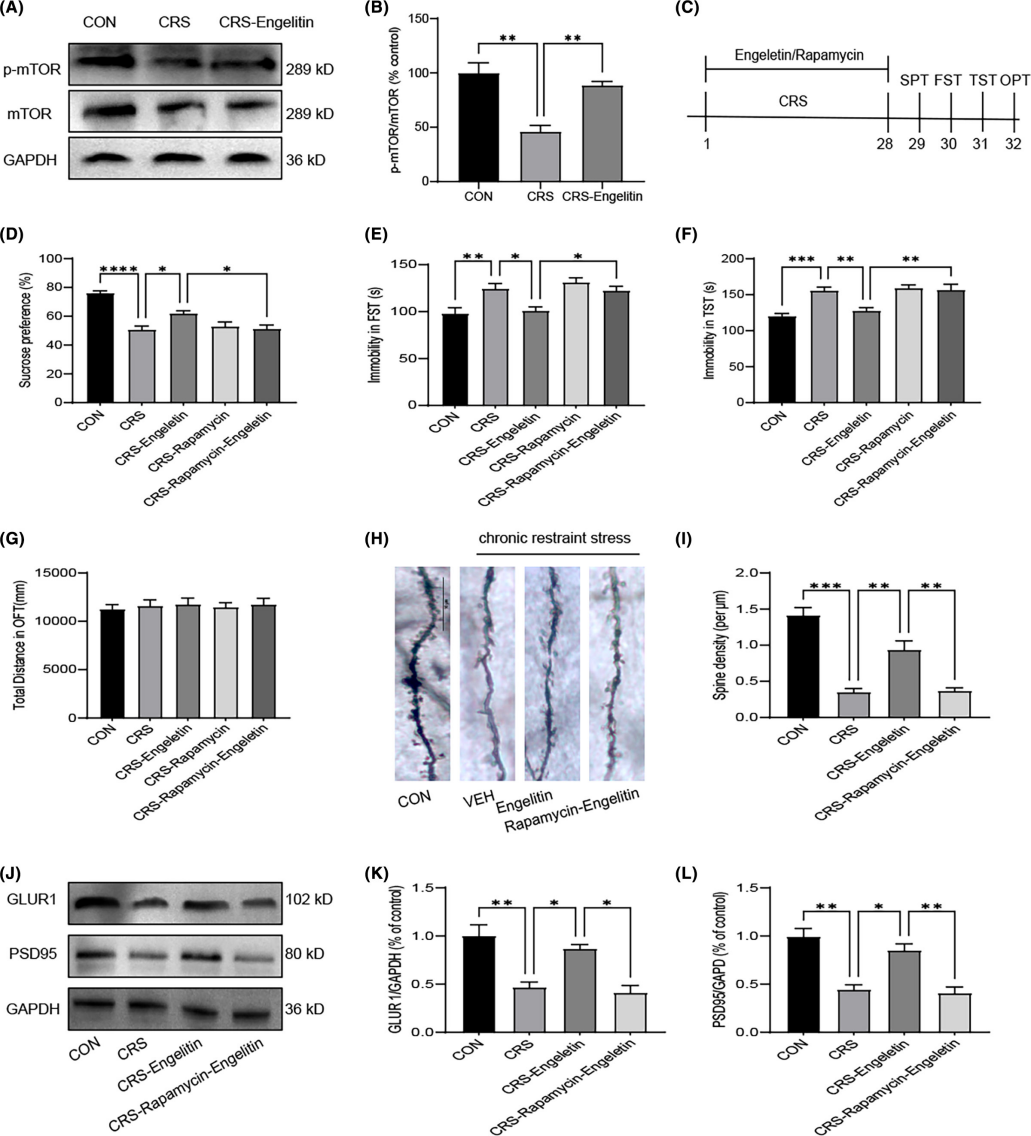
Rapamycin treatment prevents the effect of engeletin on depressant‐like depressant‐likes and synaptic plasticity in the PFC. (A, B) Western blotting indicated that the engeletin regimen reversed the CRS‐mediated reduction of PFC's p‐mTOR protein (*n* = 3). (C) Experimental protocol timeline. (D) In the CRS model, engeletin + rapamycin mice had reduced sucrose preference than engeletin mono‐treatment mice (*n* = 12). (E, F) In the CRS model, engeletin + rapamycin mice exhibited increased immobility time than engeletin mono‐treatment mice in the TST and FST (*n* = 12). (G) Neither rapamycin nor engeletin substantially affected the locomotor function of naive mice in the OFT (*n* = 12). (H, I) Golgi‐staining indicated that engeletin + rapamycin mice reversed the CRS‐mediated decrease of PFC DS density (*n* = 3). (J–L) Western blotting indicated that the engeletin + rapamycin reversed the engeletin‐mediated increase of PFCs' GLUR1 and PSD95 protein (*n* = 3). Data are expressed as mean ± SEM. *p < 0.05, **p < 0.01, ***p < 0.001, ****p < 0.0001. Two‐way anova followed by post hoc Bonferroni's test.

The critical downstream signalling pathway of BDNF‐TrkB, mTOR kinase, has been reported as the intracellular signalling mechanism linked with the efficacy of antidepressants in depressed humans and animal models.^24^ The levels of mTOR and its activated form (p‐mTOR) in the PFC of treatment groups were assessed. It was observed that compared with the CRS mice, engeletin (20 mg/kg) treatment restored the PFC p‐mTOR levels to normal (Figure 6A). A selective mTOR inhibitor was utilized to further assess if mTOR is essential for engeletin and rapamycin activity. 5 mg/kg of rapamycin (i.p., daily) was given to mice 30 min before engeletin (20 mg/kg, i.g., daily) treatment. Engeletin with rapamycin treatment suppressed the sucrose preference in the CRS‐ mice (Figure 6C). Furthermore, rapamycin treatment markedly repressed engeletin‐mediated decreased immobility period in the FST and TST (Figure 6E,F). Each group indicated no essential alterations in locomotor activity (Figure. 6G).

Additionally, whether rapamycin blocked engeletin's effects on PFC spine density and the synaptic plasticity‐linked proteins (PSD95, GLUR1) were assessed. A substantial difference between the control and CRS mice was identified. Moreover, rapamycin also inhibited engeletin's influence on spine density (Figure 6H,I), GLUR1 and PSD (Figure 6J–L) levels in the PFC. Indicating that rapamycin antagonizes the antidepressant effects of engeletin. Overall, the data indicate that mTOR signalling is essential for synaptic plasticity and engeletin's antidepressant effects.

## 5 DISCUSSION

This investigation aimed to elucidate the influence of engeletin on the depression‐like phenotype of CRS mice and to assess the potential pathways linked with BDNF signalling in the PFC. It was revealed that engeletin could ameliorate the CRS‐mediated depression‐like phenotype (including body weight loss and depressed behaviours) and protects mice's PFC synaptic ultrastructure. Furthermore, exposure of CRS mice to ANA‐12 or rapamycin inhibited the synaptic plasticity and the BDNF‐TrkB‐mTORC1 pathway in the PFC; these effects were reversed after engeletin treatment.

Basic and clinical studies demonstrate that depression is associated with reduced size of brain regions that regulate mood and cognition, including the PFC and the hippocampus, and decreased neuronal synapses in these areas.^25^, ^26^ Antidepressants can block or reverse these neuronal deficits, although typical antidepressants have limited efficacy and delayed response times of weeks to months.^27^ A notable recent discovery shows that ketamine, a N‐methyl‐D‐aspartate receptor antagonist, produces rapid (within hours) antidepressant responses in patients who are resistant to typical antidepressants.^28^, ^29^ Basic studies show that ketamine rapidly induces synaptogenesis and reverses the synaptic deficits caused by chronic stress. In addition, one of the effective therapy against depression is electroconvulsive seizure therapy, which increases synaptic plasticity by affecting synaptic connectivity and altering structure.^30^ NV‐5138 is also a recently identified synthetic leucine analog that has fast‐acting antidepressant in chronically stressed mice and is linked with enhanced synaptic protein (Synapsin‐1 and PSD‐95) expression.^31^ These findings highlight the central importance of homeostatic control of mood circuit connections and form the basis of a synaptogenic hypothesis of depression and treatment response. Here, CRS mice indicated decreased DS density and pre‐ and post‐synaptic plasticity protein markers, rescued after engeletin treatment, validating the importance of synaptic plasticity in the treatment and depression aetiology. Moreover, it was also identified that engeletin upregulated PFCs' BDNF mRNA levels in CRS mice.

The literature suggests that depression pathogenesis is closely linked with BDNF, and treatment with antidepressant drugs can effectively improve BDNF reduction due to depression, stimulating neurogenesis, increasing neural plasticity and serving as an antidepressant.^32^, ^33^ BDNF is broadly expressed in the central nervous system and regulates synapsis, memory, learning and neuroprotection. BDNF and its receptor TrkB are crucially involved in depression aetiology and antidepressant's therapeutic mechanisms.^22^, ^34^, ^35^, ^36^ Various stress procedures reduce BDNF, whereas chronic treatment with antidepressants almost always increases BDNF in the hippocampus and frontal cortex. Since bipolar and major depressive disorders indicate reduced PFCs' BDNF expression and other neurotrophic factors, antidepressant mechanism might involve elevating BDNF expression. Furthermore, infusion of ANA‐12 inhibited the antidepressant effects of engeletin, indicating that BDNF‐TrkB signalling is essential for the therapeutic action of antidepressants.

The alterations in mTOR activity were also elucidated as BDNF is its upstream regulator, and mTOR phosphorylation is an intracellular signalling mechanism that modulates antidepressant efficiency in depressed animal models and humans.^24^, ^37^, ^38^ As evaluated by the p‐mTOR‐specific antibody, the mTOR activity was notably increased in engeletin‐treated mice than in CRS mice. Furthermore, p‐mTOR levels decreased in the temporal cortex (presumably including the hippocampus) in depressed patients, and major antidepressants classes enhanced these levels and p‐mTOR function in various brain regions, including PFC. This investigation indicated that chronic engeletin treatment upregulated p‐mTOR in the PFC of stressed animals to the normal basal level of saline‐injected mice. Additionally, engeletin‐mediated antidepressant effects and increased DS were also inhibited with rapamycin co‐treatment, suggesting that the mTOR signalling pathway is important for engeletin's antidepressant and synaptic plasticity influence.

Nevertheless, our study is not without limitations. First, the pathway from BDNF to mTORC1 is complex and we targeted only one portion of this pathway. Second, engelitin only facilitates synaptic plasticity in the PFC region, while other brain regions closely related to depression, such as the hippocampus and lateral habenula, have not been involved.^39^ Hence, we could not rule out the possibility that other mechanisms in the PFC may underlie the functions of engelitin. Third, further comprehensive research for elucidating the in vivo pharmacokinetics, brain tissue distribution and in vivo toxicology of engeletin is required.

## 6 CONCLUSION

Overall, this investigation revealed that engeletin produces antidepressant‐like properties in mouse depression models, promoting synaptic plasticity by upregulating the PFCs' BDNF‐TrkB‐mTORC1 signalling pathway. This data furnishes new insight to comprehensively understand the engeletin pharmacological effects and its possible therapeutic use against major depression.

## AUTHOR CONTRIBUTIONS

**Yangyang Xu:** Data curation (equal); formal analysis (equal); funding acquisition (equal); methodology (equal); project administration (equal). **Jie Zhang:** Data curation (equal); funding acquisition (equal); investigation (equal); methodology (equal). **Linyao Yu:** Data curation (equal); methodology (equal). **Wei Zhang:** Data curation (equal); methodology (equal). **Yingtian Zhang:** Data curation (equal); methodology (equal). **Yaoqin Shi:** Data curation (equal); methodology (equal). **Shuping Zhang:** Conceptualization (equal); funding acquisition (equal); project administration (equal). **Chunmei Li:** Conceptualization (equal); data curation (equal); formal analysis (equal); funding acquisition (equal); investigation (equal); project administration (equal). **Jingwei Tian:** Conceptualization (equal); funding acquisition (equal); project administration (equal).

## CONFLICT OF INTEREST STATEMENT

There are no conflicts of interest to declare.

## Data Availability

The original contributions presented in the study are included in the article/Supplementary Material, further inquiries can be directed to the corresponding authors.
